# Nomogram for predicting mild cognitive impairment in Chinese elder CSVD patients based on Boruta algorithm

**DOI:** 10.3389/fnagi.2025.1431421

**Published:** 2025-02-03

**Authors:** Yanzi Huang, Wendie Huang, Xiaoming Ma, Guoyin Zhao, Jingwen Kang, Huajie Li, Jingwei Li, Shiying Sheng, Fengjuan Qian

**Affiliations:** ^1^Department of Neurology, The Third Affiliated Hospital of Soochow University, Changzhou, China; ^2^Department of Neurology of Suzhou Hospital, Affiliated Hospital of Medical School, Nanjing University, Suzhou, China; ^3^Department of Neurology of Drum Tower Hospital, Medical School and the State Key Laboratory of Pharmaceutical Biotechnology, Nanjing University, Nanjing, China; ^4^Institute of Brain Sciences, Nanjing University, Nanjing, China; ^5^Jiangsu Key Laboratory for Molecular Medicine, Medical School of Nanjing University, Nanjing, China; ^6^Jiangsu Province Stroke Center for Diagnosis and Therapy, Nanjing, China; ^7^Nanjing Neurology Clinic Medical Center, Nanjing, China; ^8^Department of Endocrinology, The Third Affiliated Hospital of Soochow University, Changzhou, China

**Keywords:** MCI, CSVD, IGF-1, cognition, cognitive impairment, stroke, nomogram

## Abstract

**Background:**

The number of patients with cerebral small vessel disease is increasing, especially among the elderly population. With the continuous improvement of detection techniques, the positivity rate keeps increasing. Our goal is to develop a nomogram for early identification of PSCI and PSCID in stroke patients.

**Methods:**

In a retrospective cohort, chained data imputation was performed to ensure no statistical differences from the original dataset. Subsequently, Boruta algorithm was utilized for variable selection based on their importance, followed by logistic regression employing backward stepwise regression. Finally, the regression results were visualized as a Nomogram.

**Results:**

The nomogram chart in this study achieves clinical utility in a concise and user-friendly manner, passing the Hosmer-Lemeshow goodness-of-fit test. ROC and calibration curves indicate its high discriminative ability.

**Conclusion:**

While CSVD is prevalent among middle-aged and older individuals, cognitive decline trajectories differ. Endocrine metabolic indicators like IGF-1 offer early predictive value. This study has produced a succinct nomogram integrating demographic and clinical indicators for medical application.

## 1 Introduction

Ischemic strokes are notably common among the elderly. Although current research results indicate that cerebral small vessel disease (CSVD) has lower disability and mortality rates, and relatively mild structural and functional damage to the brain’s neural architecture, statistics show that CSVD patients have a significantly higher stroke recurrence rate compared to large vessel brain diseases. CSVD (Petty et al., 2000) is characterized by clinical, cognitive, and pathological features resulting from structural or functional changes in various components of small vessels in the brain, including small arteries, arterioles, capillaries, and venules. With the aging population trend and rapid advancements in magnetic resonance imaging technology, the incidence and detection rates of CSVD are increasing. Specifically, white matter hyperintensities are highly prevalent in the aging population, observed in 80% of healthy individuals at the age of 60 and nearly all healthy individuals at the age of 90 (de Leeuw et al., 2001). The primary clinical manifestations of cerebral small vessel disease include stroke, cognitive decline, dementia, psychiatric symptoms, gait abnormalities, and urinary incontinence. Furthermore, cognitive impairment is the main complication of cerebral small vessel disease (Pantoni, 2010), imposing a heavy economic burden on families, healthcare systems, and society as a whole. Cognitive impairment and dementia following stroke (Rost et al., 2022) serve as crucial factors contributing to morbidity and mortality among stroke patients. Early identification of post-stroke cognitive impairment facilitates timely implementation of intervention measures and helps prevent recurrent strokes. Previous study (Weaver et al., 2021) have indicated that approximately half of individuals experience post-stroke cognitive impairment (PSCI) within the first year following a stroke. Infarct location is a potential determinant of PSCI, yet strategic infarct locations predictive of PSCI remain elusive. Our aim is to achieve early identification of PSCI and post-stroke cognitive impairment with dementia (PSCID) in stroke patients through the use of the blood biomarker insulin-like growth factor-1 (IGF-1).

More importantly, IGF-1 participating in many physiological metabolic processes throughout the body, IGF-1 plays a crucial role in growth, development, lifespan control, and aging processes. Under physiological conditions, IGF-1 can be widely expressed in the central nervous system (Holly and Perks, 2012). Research findings suggest that IGF-1 is associated with the risk of ischemic stroke, and low circulating IGF-1 may be a particularly important determinant of ischemic stroke events in obese and diabetic patients. Serum IGF-1 levels may serve as important biomarkers for assessing the risk of ischemic diseases, especially in individuals with type 2 diabetes or central obesity (Saber et al., 2017). Increasing evidence indicates that IGF-1 plays a critical role in neurogenesis and is associated with cognitive impairment (Holly and Perks, 2012; Saber et al., 2017; Werner and LeRoith, 2014; Vagelatos and Eslick, 2013). It can maintain homeostasis in patients with high blood sugar, improve insulin resistance, alleviate Aβ protein-induced brain tissue damage, inhibit abnormal phosphorylation of tau protein, reduce neurotoxicity of NO, protect neurons, improve brain ischemia and hypoxia, thereby reducing the risk of cognitive impairment (Piriz et al., 2011; Hölscher, 2014; Craft and Watson, 2004).

In this study, Boruta Algorithm was applied to select the variables. As a popular algorithm, it likely involves solving complex problems through iterative steps to improve a solution, which starts with initial inputs, refines them through repeated calculations, and stops when a satisfactory result is reached. The variables been selected would develop a multivariate logistic regression. After step back method, the final model will be calibrated and validated.

## 2 Materials and methods

### 2.1 Study patients

Study patients with stroke were continuously enrolled in the Department of Neurology, The Third Affiliated Hospital of Soochow University from November, 2019 to August 2020.

The patient underwent clinical assessment by the physician and was diagnosed with CSVD (Hayes et al., 2021) following magnetic resonance imaging (MRI) examination. The most common manifestations of CSVD include White Matter Hyperintensity (WMH) and Lacunar Infarction (LI). WMH appears as high signal intensity on T2-weighted sequences and as iso-or hypointense signals on T1-weighted sequences (though not as hypointense as cerebrospinal fluid). Lesions manifest as white matter attenuation or low density on computed tomography (CT) scans. LI is defined as circular or oval lesions with diameters ranging from 3 to 15 mm, detected on both T1-weighted and T2-weighted images, with surrounding halos (Hayes et al., 2021; Baessler et al., 2018) evident on Fluid Attenuation Inversion Recovery (FLAIR) images.

Additionally, the requirement for informed consent from the study patients was waived due to the retrospective study design.

#### 2.1.1 Patients were included in the study if they met the following inclusion criteria

1. Patients aged between 50 and 85 years with good physical condition and ability to cooperate with examinations.
2. Patients diagnosed with CSVD based on the imaging diagnostic criteria established by the International Study of CSVD Imaging Standards group in 2013, in conjunction with the imaging criteria outlined in the 2015 “Chinese Consensus on Diagnosis and Treatment of Cerebral Small Vessel Disease” were recruited.

#### 2.1.2 Exclusion criteria for the study were as follows

1. Presence of severe visual, auditory, or motor impairments affecting cognitive function testing.
2. History of other endocrine disorders such as severe hypoglycemia or ketoacidosis, hyperthyroidism, hypothyroidism, systemic lupus erythematosus, etc.
3. History of diseases or central nervous system injuries causing cognitive impairment, such as tumors, severe hepatic or renal dysfunction, cranial trauma, congenital intellectual disability, toxic encephalopathy, alcohol or substance dependence, psychiatric disorders, neurodegenerative diseases, neurosyphilis, epilepsy, etc.
4. History of other dementia before the diagnosis of CSVD, such as Alzheimer’s disease, Lewy body dementia, frontotemporal dementia, mixed dementia, etc.

### 2.2 Data collection

To conduct a baseline medical history investigation for all recruited patients, each study subject was interviewed by the investigator using a standardized questionnaire to fill out a form.

#### 2.2.1 Demographic and medical history

Demographic data including gender, age, years of education, history of diabetes (defined as fasting blood glucose level > 126 mg/dL on multiple occasions or at any time, or the use of anti-diabetic medication, with blood glucose level > 200 mg/dL), and history of stroke (clinically evident previous stroke or diagnosis by cranial MRI) were collected. Upon admission, systolic and diastolic blood pressure measurements were taken. Fasting venous blood samples were drawn from enrolled patients at 8:00 am the following day to measure levels of triglycerides, total cholesterol, low-density lipoprotein cholesterol, fasting blood glucose, HbA1c, fasting insulin, C-peptide, and anti-insulin antibodies. All blood analyses were conducted in the standard laboratory of the Third Affiliated Hospital of Soochow University.

#### 2.2.2 The assay method for serum insulin-like growth factor-1

On the following morning at 8:00 am, 3 mL of fasting venous blood was drawn from enrolled patients’ elbow veins. The blood samples were centrifuged at 4°C at a speed of 4,000 rpm for 20 min, and the serum samples were stored at-80°C until further downstream analysis. Serum IGF-1 concentrations were quantified using an IGF-1 ELISA assay kit, measuring absorbance at 450 nm. IGF-1 levels (ng/ml) were calculated according to the manufacturer’s instructions.

### 2.3 Cognition assessment

The Montreal Cognitive Assessment (MoCA), a more sensitive tool than the Mini-Mental State Examination (MMSE), is used to detect cognitive impairment in patients more effectively. This enables early identification and intervention for mild cognitive impairment patients, thereby improving their quality of life. The MoCA assessment includes the following domains: 1. Visuospatial and Executive Functioning; 2. Naming; 3. Immediate and Delayed Recall; 4. Attention and Calculation; 5. Language; 6. Abstraction; 7. Orientation, which encompasses time and place orientation. The maximum score is 30 points, with an additional point added if the years of education are less than 12. A score of ≥26 is considered normal, indicating no cognitive impairment. Lower scores indicate greater cognitive decline.

### 2.4 Statistical analysis

Firstly, utilized multiple imputation techniques were applied to fill in variables unrelated to the outcome. Secondly, concerning the significant imbalance in MCI outcomes, with only 50 patients without MCI and 166 patients with MCI. Hence, Synthetic Minority Over-sampling Technique for Nominal and Continuous data technique was employed to balance the sample size. Table 1 was constructed based on the result of dataset preprocessing to illustrate the differences in baseline characteristics. Subsequently, Boruta variable selection technique was applied on the development set. Following this, logistic regression was fitted on the development set with backward regression to ensure the lowest AIC. Finally, the model was calibrated and validated in the validation set.

**Table 1:** Demographic and cognitive characteristics of the study population after applying SMOTE-NC technique (*n* = 332).

Characteristic	CI (*n* = 166)	NCI (*n* = 166)	All (*n* = 332)	*p*-value
Age (years)	69.30 ± 8.38	64.03 ± 7.36	68.57 ± 8.73	<0.001
Education years	8.87 ± 2.49	9.38 ± 1.56	9.12 ± 2.09	0.025
HbA1c (mmol/L)	6.86 ± 1.76	5.96 ± 0.85	6.41 ± 1.45	<0.001
Systolic blood pressure (mmHg)	148.01 ± 20.59	144.05 ± 17.38	146.03 ± 19.13	0.060
Diastolic blood pressure (mmHg)	85.54 ± 11.68	84.57 ± 9.02	85.05 ± 10.43	0.400
Insulin (pmol/L)	62.80 ± 41.54	47.82 ± 24.38	55.31 ± 34.82	<0.001
C-Peptide (pmol/L)	873.16 ± 396.05	731.66 ± 217.29	802.41 ± 326.72	<0.001
Anti-insulin antibodies (IU/ml)	4.72 ± 2.77	3.83 ± 1.65	4.28 ± 2.32	<0.001
IGF-1 (ng/ml)	114.11 ± 28.99	147.67 ± 38.61	130.89 ± 38.00	<0.001
FBG (mmol/L)	6.52 ± 2.49	5.58 ± 1.55	6.05 ± 2.12	<0.001
Total cholesterol (mmol/L)	4.51 ± 1.20	4.34 ± 1.00	4.43 ± 1.11	0.152
Triglyceride (mmol/L)	1.79 ± 1.13	1.54 ± 0.86	1.66 ± 1.01	0.026
Low-density lipoprotein (mmol/L)	2.68 ± 1.02	2.66 ± 0.76	2.67 ± 0.90	0.859
Sex (%)				0.296
Male	115 (69.28)	105 (63.25)	220 (66.27)	
Female	51 (30.72)	61 (36.75)	112 (33.73)	
History of diabetes mellitus (%)				<0.001
Yes	102 (61.45)	23 (13.86)	125 (37.65)	
No	64 (38.55)	143 (86.14)	207 (62.35)	
White matter lesions (%)				<0.001
Yes	46 (27.71)	19 (11.45)	65 (19.58)	
No	120 (72.29)	147 (88.55)	267 (80.42)	

CI, cognitive impairment; NCI, non-cognitive impairment; FBG, fasting blood glucose.

All statistical tests were two-sided, with significance set at a *p*-value <0.05. Continuous data were presented as mean ± SD, while categorical data were represented as proportions. All statistical analyses were performed using R 4.2.3.

## 3 Results

### 3.1 Demographic characteristics and clinical information

See Table 1 for details.

### 3.2 Dataset division

The dataset was randomly divided into two groups, with no statistically significant differences observed between the development and validation groups across various indicators.

### 3.3 Boruta algorithm variable selection

The Boruta technique is a feature selection algorithm evolved from Random Forest. Its’ function is to identify all features correlated with the responding variable and rank them based on their importance. The results obtained in this study were as follows: IGF-1, History of Diabetes Mellitus, HbA1c, Insulin, Education Years, SBP, C-Peptide, DBP, Age, Total Cholesterol, Anti-Insulin Antibody, FBG, LDL-C, Triglyceride, White Matter Lesion (WML), and Sex.

### 3.4 Logistic regression

**Figure 1:**
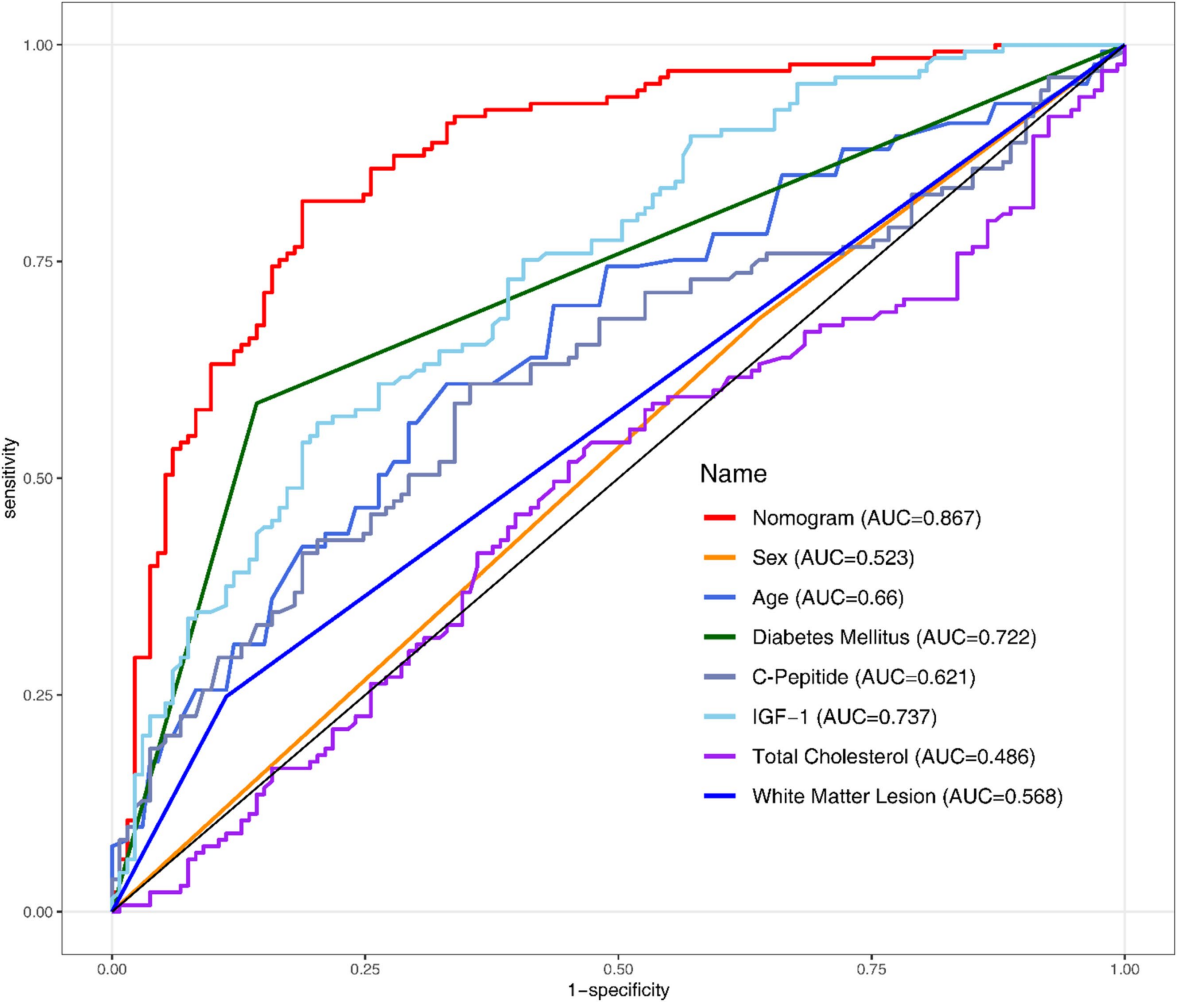
ROC curve of the development set.

**Figure 2:**
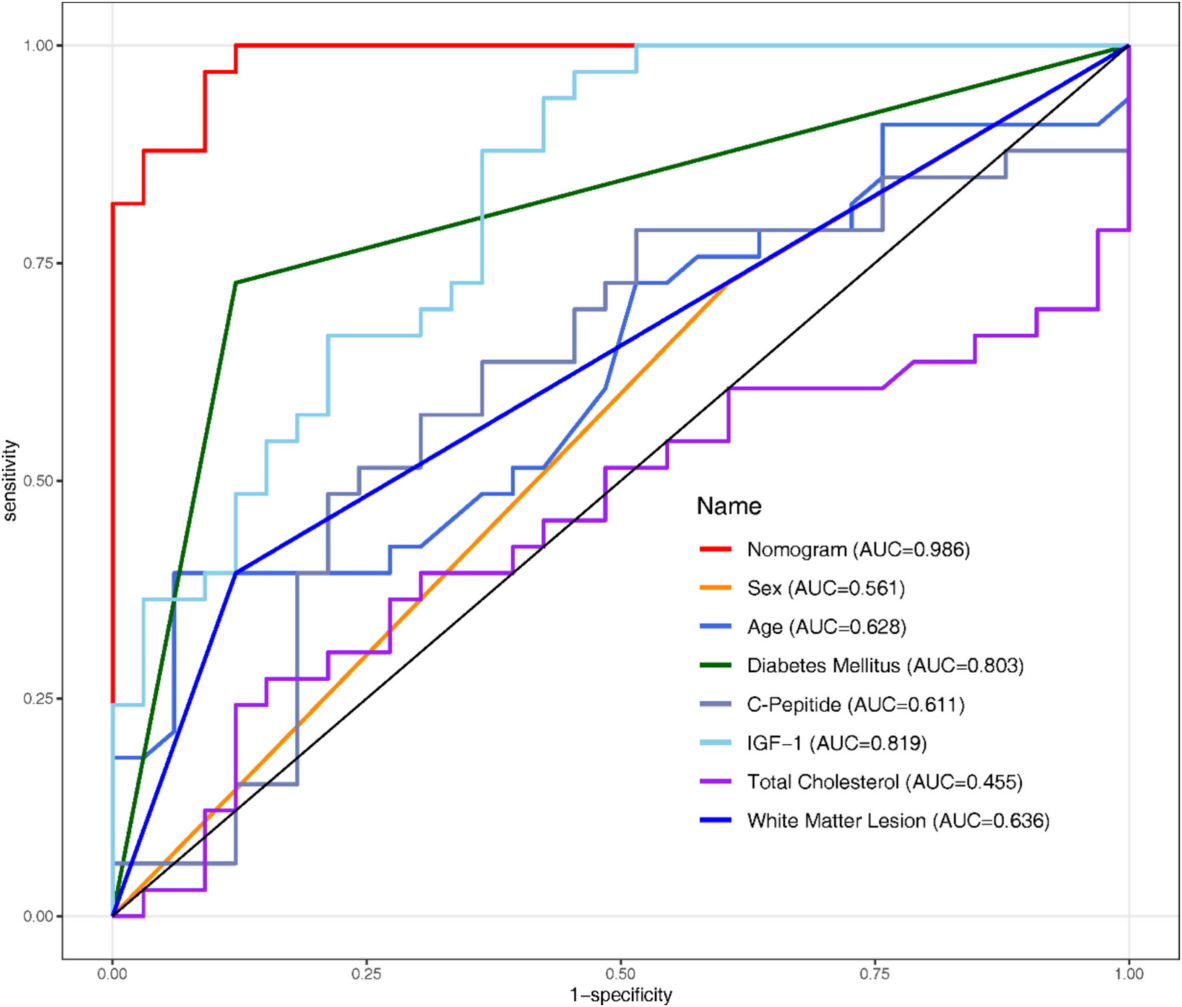
ROC curve of the validation set.

All the variables mentioned in section 3.3 were analysed using Univariate and Multivariate Logistic Regression (Table 2). Following the original regression, a backward stepwise regression was performed to lowering the AIC from 277.97 to 265.42, intending to maintain high predictive performance while streamlining the model. After establishing the regression model, Receiver Operating Characteristic (ROC) curves and the Area Under the Curve (AUC) were employed to evaluate the discriminative performance of both the development (Figure 1) and validation datasets (Figure 2).

**Table 2:** Univariate and multivariate logistic regression of the seven predictors.

Variables	Univariate analysis		*p*-value	Multivariate analysis		*p*-value	VIF
	OR	95% CI		OR	95% CI		
Sex							1.118
Female	1.00			1.00			
Male	0.82	0.49–1.36	0.437	0.48	0.24–0.96	0.041	
Age	1.07	1.04–1.11	<0.001	1.03	0.99–1.08	0.108	1.140
History of diabetes mellitus							1.173
No	1.00			1.00			
Yes	8.51	4.77–15.78	<0.001	7.11	3.58–14.85	<0.001	
C-Peptide	1.00	1.00–1.00	<0.001	1.00	1.00–1.00	0.074	1.168
IGF-1	0.97	0.97–0.98	<0.001	0.97	0.96–0.98	<0.001	1.252
Total cholesterol (mmol/L)	1.12	0.90–1.40	0.303	1.34	1.00–1.85	0.059	1.068
WML						0.042	1.081
No	1.00			1.00			
Yes	2.60	1.36–5.17	0.005	2.42	1.05–5.77		

Additionally, the collinearity among variables is assessed using variance inflation factors (VIF). VIF <10 implies that there are no statistically significant correlations among the variables in the model.

Meanwhile, The Hosmer-Lemeshow Test and calibration curve were conducted to assess the adequacy of calibration.

### 3.5 Nomogram development

**Figure 3:**
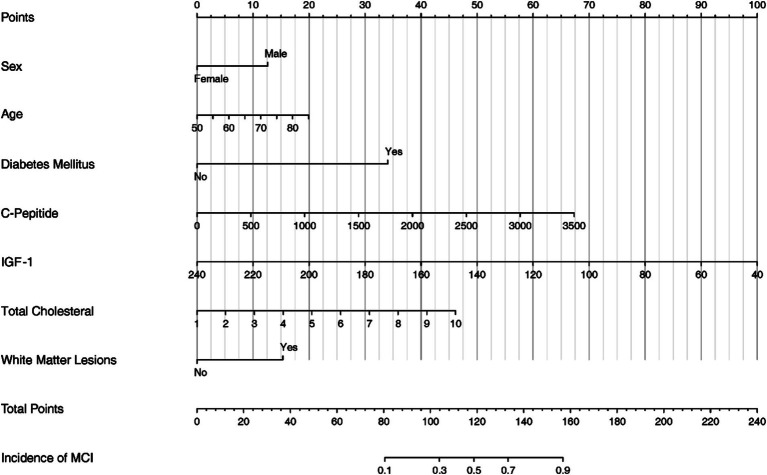
The nomogram based on the final logistic regression model.

The nomogram serves as a visual representation of the established Logistic Regression model (Figure 3).

**Figure 4:**
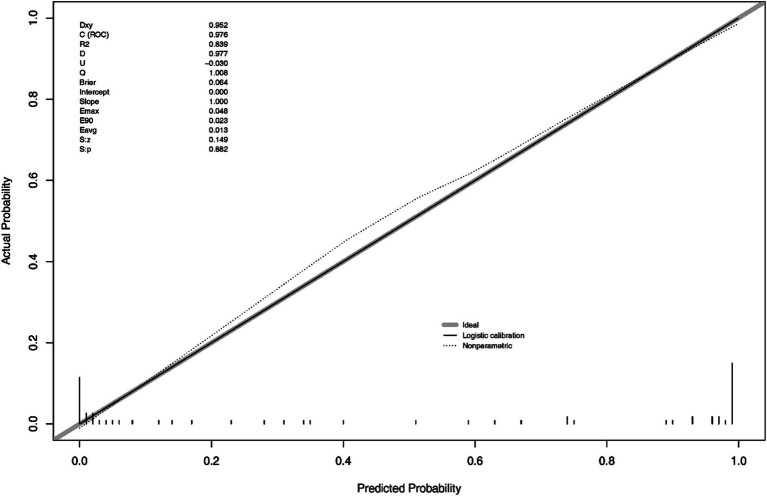
Calibration curve for the nomogram model in the validation set.

In the Figure 4, S: *p* > 0.005 indicates that the calibration of the model passed the goodness-of-fit test, as did the result of Hosmer-Lemeshow test (χ^2^ = 1.444, *p*-value = 0.9936), indicating the calibration curve demonstrated good agreement between predicted and observed outcomes.

### 3.6 Clinical practice

**Figure 5:**
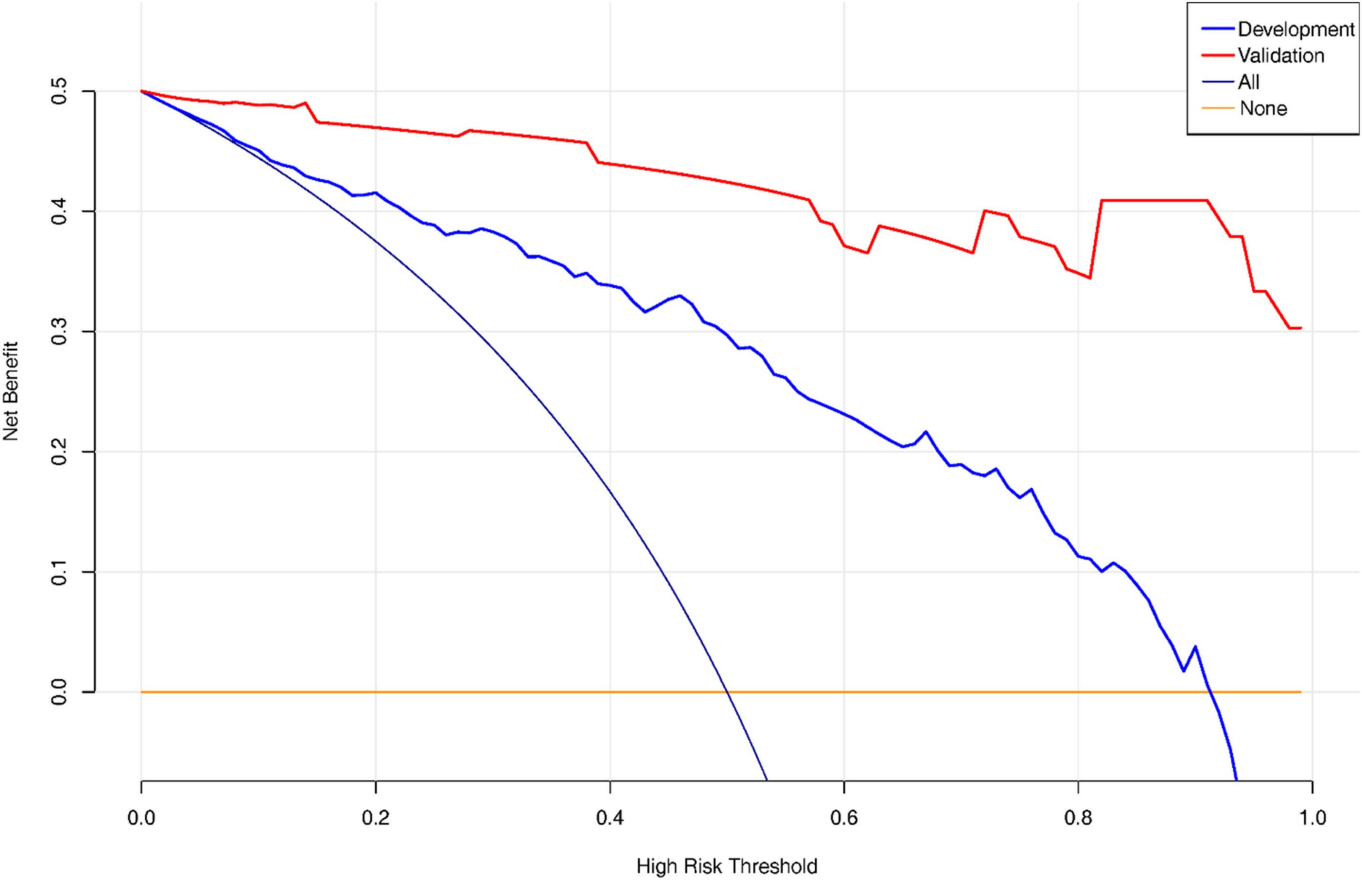
DCA of the nomogram model in the development and validation set.

Decision Curve Analysis (DCA) curve was plotted to showcase the clinical net benefit of the nomogram (Figure 5), with a focus on the validation set, which implies that the nomogram model demonstrates a clinical net benefit across all thresholds.

## 4 Discussion

This study developed a nomogram based on demographic and clinical indicators, demonstrating high accuracy in predicting MCI in elderly patients with CSVD. CSVD is a pathological process affecting brain microvessels, leading to functional impairment in the elderly population, characterized mainly by cognitive impairment and gait decline (Kong et al., 2024). Therefore, this study developed a nomogram to predict the risk of MCI in in Chinese older CSVD patients. The nomogram includes the following seven variables: IGF-1, C-Peptide, age, diabetes mellitus, total cholesterol, WML, sex.

Previous studies (Mäntyselkä et al., 2019; Talbot et al., 2012) have indicated that endocrine metabolic indicators like IGF-1 are linked to cognitive function. Our study extends these findings by integrating IGF-1 with other clinical variables to improve predictive accuracy. IGF-1 is a single chain peptide (7.5 kDa) involved in many physiological, anabolic and metabolic processes throughout the body. It is considered to be a major homeostatic regulator, which plays a role in growth, development, life control and aging, and plays an important role in normal brain development (Frater et al., 2018). IGF-1 is neurotrophic and can promote neuronal survival by inhibiting apoptosis. It has also been proved in animal model experiments that IGF-1 enhances learning and memory processes, a phenomenon observed in human as well. In AD patients, there is evidence that the expression of IGF signaling pathway components is decreased, which indicates that IGF-1 is a protective factor of cognitive function (Bishop et al., 2010). IGF-1 receptor (IGF-1R) protein expression is concentrated in specific brain regions including hippocampus, amygdala, thalamus, hypothalamus, choroid plexus and parahippocampal gyrus (Calvo et al., 2013). These brain regions are associated with emotion, cognition and memory, supporting the idea that reducing IGF-1, which leads to impaired function in these areas, may lead to memory disorders and cognitive impairment that are common in aging. All these prove that IGF-1 as an endocrine and metabolic index has a certain value in early prediction of cognitive decline. However, there is no definite evidence to prove whether increasing the concentration of circulation or brain IGF-1 can slow down or even reverse the occurrence of cognitive impairment, which needs further exploration and research to provide effective guidance for follow-up intervention measures of cognitive impairment.

C-peptide is a by-product of insulin synthesis, and it is broken down along with insulin from precursor proteins in the pancreas (i.e., proinsulin). C-peptide plays a role in the regulation of insulin release, but its main function is as a physiological indicator to evaluate the level of insulin secretion. There is still some controversy about the relationship between C-peptide and cognitive impairment, but some studies have suggested that C-peptide may be related to the decline of cognitive function. Studies have found that patients with declining cognition exhibited baseline hyperinsulinemia and elevated plasma c-peptide levels with normal c-peptide/insulin ratios, suggesting that insulin production was increased, but insulin clearance was normal (Khuder et al., 2019). In addition, some other studies have also found that C-peptide may be involved in the development of neurodegenerative diseases. Basic science research has linked deficiencies in insulin breakdown to AD pathology. Many studies have also demonstrated an association between AD and insulin metabolism disorders (Okereke et al., 2009; de la Monte et al., 2019). One study found that both serum and cerebrospinal fluid levels of C-peptide in MCI/AD were higher (de la Monte et al., 2019). This suggests that C-peptide may somehow be involved in the development of cognitive impairment and neurodegenerative diseases. Although the specific mechanism is not completely clear, there is a certain correlation between the level of C-peptide and cognitive function, which provides some clues for exploring the pathogenesis and prevention of cognitive impairment.

Diabetes, as a systemic metabolic disease, has long been considered as an important risk factor for cognitive impairment and dementia. Studies have shown that patients with diabetes have hyperglycemia, insulin resistance and inflammation, which can damage cerebrovascular health and/or directly affect neuronal function through different mechanisms, and then affect cognitive ability (Biessels et al., 2006; Crane et al., 2013). More specifically, the association between diabetes and stroke is of particular concern because both are common pathological conditions leading to cerebrovascular injury. PSCI is a common complication in stroke survivors, and diabetes, as an important risk factor for stroke, may further aggravate the occurrence of PSCI (Iadecola et al., 2019). Under the premise of diagnosis of CSVD, diabetes may aggravate the decline of cognitive function by accelerating the progression of microangiopathy. This study suggests that the presence of diabetes in elderly patients with CSVD may indicate a greater risk of cognitive impairment. Therefore, early identification and management of diabetic patients in this group is of great significance to prevent or slow down the decline of cognitive function.

WMH is a common imaging manifestation of CSVD, especially in the elderly. CSVD is closely related to the decline of cognitive function. The results show that there is a correlation between the existence of WMH and the decline of cognitive function. Many research teams have obtained the same results in the past, and there is a positive correlation between the degree of WMH and the severity of cognitive decline in the elderly (de Leeuw et al., 2001). CSVD is one of the important causes of cognitive decline, in which WMH is considered to be one of the important signs of the occurrence and development of the disease (Pantoni, 2010). In addition, cognitive impairment and dementia after stroke are common complications, and CSVD is one of the important potential factors (Rost et al., 2022). This finding further supports the link between WMH and cognitive decline.

Total cholesterol is an important lipid in the human body, which has an important effect on the function of the nervous system. Some long-term epidemiological studies have found that high total cholesterol levels are associated with decreased cognitive function and an increased incidence of AD (Anstey et al., 2008; Solomon et al., 2009). These studies looked at large populations and found a link between high cholesterol levels and the progression of cognitive decline. However, some studies have come to the opposite conclusion that low cholesterol levels are associated with cognitive decline (Reitz et al., 2010). This opposite view may be related to different research methods, sample selection and research objects. Generally speaking, the relationship between total cholesterol and cognitive function is complex. High cholesterol levels may be related to the occurrence of cognitive impairment, but low cholesterol levels may also have a similar correlation. Despite some controversy, most studies support a link between high total cholesterol levels and an increased risk of cognitive impairment, which is consistent with our results. Further research still needs to explore this relationship in depth.

The positive relationship between aging and cognitive impairment is almost recognized, and the prevalence of MCI increases with age (Li et al., 2020). And age is a non-intervention factor, so the cognitive impairment of the elderly is particularly worthy of attention, which is also the significance of our development and prediction of the risk of cognitive impairment in the elderly.

The relationship between gender and cognitive impairment is a complex and changeable topic. Some studies have shown that gender may play an important role in the occurrence and development of cognitive impairment. Some early studies have suggested that women are more likely to develop cognitive impairment, especially AD. This view is partly based on the fact that women have a longer life expectancy, and they are more likely to face the risk of cognitive impairment if they are known to have a positive correlation between age and cognitive impairment. But there is growing evidence that men also face considerable risks. Although women generally live longer than men, once factors such as age and education are controlled, gender may have less significant impact on the risk of cognitive decline, but men may have a higher risk of cognitive impairment in later years (Gao et al., 1998). This may be related to the fact that men are more vulnerable to some cardiovascular diseases, and there is a close link between cardiovascular health problems and cognitive impairment. Although the impact of gender on the occurrence of cognitive impairment is complex, there is growing evidence that men may be at higher risk in some ways. A study on the development of a risk prediction model for the occurrence of MCI in the elderly has obtained the same results as this study (Ma et al., 2023), that is, men have a higher risk of developing MCI than women. The relationship between gender and cognitive impairment is affected by a variety of factors, including longevity, biological factors and socio-economic factors. Research in this field is still developing, and more research is needed to explore the impact of gender on cognitive impairment.

Our nomogram integrates various prognostic variables to estimate individual probabilities of cognitive impairment in older CSVD patients. It included IGF-1, C-Peptide, age, diabetes mellitus, total cholesterol, White Matter Lesion, sex. Some of these indicators can be obtained directly, while others are clinical indicators and imaging features. For example, an 85-year-old CSVD male with no history of diabetes, clinical tests showed that C-peptide was 500 pmol/L, IGF-1 was 160 ng/mL, total cholesterol was 4.5 mmol/L, and imaging examination showed WMH. According to the nomogram, the total score would be 115 (20 for age, 12.5 for sex, 0 for diabetes, 10 for C-peptide, 40 for IGF-1, 17.5 for total cholesterol, and 15 for WMH). This score corresponds to a predicted risk of MCI of about 40%. A larger cohort study is needed to further explore the seven results proposed by our study. Other variables not included in the final regression model but which have the predictive value of statistical differences within the studied population, such as fasting blood glucose, HbA1c, fasting insulin, and anti-insulin antibodies, should also be explored. All in all, the nomogram can be easily implemented in clinical settings to identify high-risk patients early, allowing for timely interventions to prevent or mitigate cognitive decline.

## 5 Conclusion

Although CSVD is common among middle-aged and older individuals, the trajectory of cognitive decline varies among patients. Our study developed a concise and user-friendly nomogram that integrates demographic and clinical indicators, providing early predictive value for cognitive impairment. This tool holds promise for enhancing clinical decision-making and improving patient outcomes through tailored interventions. Further research is warranted to validate these findings and explore additional biomarkers that may enhance predictive accuracy.

## Data Availability

The original contributions presented in the study are included in the article/supplementary material, further inquiries can be directed to the corresponding authors.
